# IL-4-mediated monocyte differentiation modulates CD163 expression and PRRSV infection

**DOI:** 10.3389/fmicb.2026.1817131

**Published:** 2026-06-04

**Authors:** Narae Kim, Young-Jun Ju, Yoon-Chul Kye, Cheol Gyun Kim, Young Jin Pyung, Da-Jeong Park, Sun-Jong You, Hakhyun Ka, Seung Hyun Han, Cheol-Heui Yun

**Affiliations:** 1 Department of Agricultural Biotechnology, Research Institute of Agriculture and Life Sciences, Seoul National University, Seoul, Republic of Korea; 2 AN R&D Center, CJ Feed and Care, Seoul, Republic of Korea; 3 Division of Biological Science and Technology, Yonsei University, Wonju, Republic of Korea; 4 Department of Oral Microbiology and Immunology, Dental Research Institute, School of Dentistry, Seoul National University, Seoul, Republic of Korea; 5 Institutes of Green-Bio Science and Technology, Seoul National University, Pyeongchang, Republic of Korea; 6 Center for Food and Bioconvergence, Seoul National University, Seoul, Republic of Korea

**Keywords:** CD163, CD8^+^ T cell, interleukin-4 (IL-4), monocyte derived cell (MDC), porcine reproductive and respiratory syndrome virus (PRRSV)

## Abstract

Host immune status critically influences susceptibility to viral infections; however, the impact of cytokine-driven innate immune differentiation on viral permissiveness remains poorly understood. Here, we investigated whether IL-4-mediated monocyte differentiation modulates porcine reproductive and respiratory syndrome virus (PRRSV) infection. Porcine monocytes were differentiated in the presence of GM-CSF with or without IL-4 to generate monocyte-derived cells (MDCs). Notably, PRRSV replication was significantly higher in MDCs generated without IL-4 compared to those differentiated in the presence of IL-4, which was strongly associated with increased CD163 expression. Consistently, CD163 expression was downregulated in an IL-4-dependent manner. Following PRRSV infection, MDCs differentiated without IL-4 exhibited reduced TNF-*α* production but elevated IL-6 and IL-10 levels. In addition, MDCs generated without IL-4 showed increased expression of CD80 and SLA-1 and significantly impaired the proliferation of autologous CD8^+^ T cells compared to MDCs differentiated with IL-4. *In vivo* analysis further revealed a negative correlation between plasma IL-4 levels and CD163 expression in alveolar MDCs of PRRSV-infected pigs, which was associated with increased proportions of CD8^+^ T cells. Collectively, these findings suggest that IL-4-mediated innate immune programming regulates cellular permissiveness to PRRSV infection and may contribute to protective host immune responses.

## Introduction

Porcine reproductive and respiratory syndrome (PRRS) is a viral disease that causes reproductive and respiratory disorders in pigs. PRRS virus (PRRSV) is a major pathogen that threatens the pig production industry worldwide (Renken et al., 2021). When sows are infected with PRRSV, the virus can cause reproductive problems that result in abortion and stillbirth, as well as serious respiratory diseases in piglets with weakened immune systems (Lunney et al., 2016). Because of viral mutations, the highly pathogenic PRRSV strain that appeared in China in the late 2000s caused the mortality rate to reach 20%, which led to significant economic loss and social impacts worldwide (Tian et al., 2007).

A hallmark of PRRSV is its limited cellular tropism, which is confined to the monocyte/ macrophage lineage (Duan et al., 1997). As the primary cellular target of PRRSV, porcine alveolar macrophages have been extensively studied during PRRSV infection. In contrast, recent studies of PRRSV infection involving dendritic cells (DCs), which share the myeloid lineage with macrophages, have been inconclusive because of differences regarding *in vitro* DCs generation methods and inherent variability in the virus. Additionally, although monocyte-derived macrophages (moMacs) are sensitive to the virus, little is known regarding the susceptivity of monocyte-derived DCs (moDCs) (Loving et al., 2015). Therefore, although it is clear that mononuclear phagocytic cells display differences in PRRSV susceptivity, the mechanisms by which these differences occur remain unknown.

Monocyte-derived cells (MDCs), such as moMacs and moDCs, are differentiated from bloodderived monocytes that have been treated *in vitro* with macrophage colony-stimulating factor (M-CSF), granulocyte-macrophage colony-stimulating factor (GM-CSF), or GM-CSF together with interleukin-4 (IL-4); these cells could serve as useful research tools for studying DCs and macrophages (Bautista et al., 2007; Franzoni et al., 2017). The results of previous studies have suggested that moMacs differentiated with M-CSF or GM-CSF exhibit high PRRSV susceptivity (Franzoni et al., 2017; Gao et al., 2018). However, in moDCs differentiated with GM-CSF and IL-4, the susceptivity is less clear; for example, viral titer varied according to kinetic differences influenced by multiplicity of infection (MOI), PRRSV strain, and moDCs maturation status (Singleton et al., 2016; Bordet et al., 2018a). It is likely that there are differences in PRRSV sensitivity among these cells. However, few studies have examined the effects of growth factors and cytokines required for MDC differentiation on PRRSV sensitivity in pigs. Additionally, most studies thus far have been conducted using cell lines, rather than primary cells.

IL-4, which is involved in the differentiation of moDCs, is significantly upregulated in the blood of PRRSV-infected pigs (Dwivedi et al., 2012). In pigs with respiratory disease, IL-4 has inhibitory effects on inflammatory cytokines and alveolar macrophage activation; however, its role in PRRSV infection is unclear. In humans, MDCs differentiated with GM-CSF possess both macrophage and DC properties; however, the addition of IL-4 caused the cells to lose their macrophage characteristics during the acquisition of DC properties (Sander et al., 2017). Additionally, there are functional differences (e.g., uptake ability, cell migration, and metabolic changes) associated with IL-4 and the MDCs differentiation period (Sander et al., 2017). Therefore, the present study investigated the effects of IL-4 on the functional characteristics of PRRSV-infected MDCs during the differentiation process. Specifically, we hypothesized that the IL-4-mediated regulation of MDCs differentiation in pigs affects MDCs functionality and may affect the immune response to PRRSV infection.

## Materials and methods

### Cell culture

Approved porcine blood samples (IACUC no. SNU-150327-2) were obtained from 4to-6-month-old Landrace–Yorkshire–Duroc pigs from Hyupsin Food Co., Ltd. (Gyeonggi-do, Korea). Porcine peripheral blood mononuclear cells (pPBMCs) were isolated as previously described (Kim et al., 2022; Bøyum et al., 2002). Freshly isolated PBMCs were incubated with anti-human CD14 (clone Tuk4) microbeads for 15 min at 4 °C. The cell suspension was applied to an LS column using a Quadro-MACS system (Miltenyi Biotec, Germany). Sorted CD14 + monocytes were seeded in medium supplemented with 10 ng/mL of recombinant porcine GM-CSF, with or without 25 ng/mL recombinant porcine IL-4 (R&D Systems, USA) for 4 days at 37 °C in an incubator with 5% CO_2_. MARC-145 cells were cultured in high glucose DMEM containing 5% FBS (Gibco, USA), 1% sodium pyruvate, 2% heparin sulfate proteoglycan 2, and 1% antibiotics.

### Viral propagation and titration

PRRSV, strain CP07-401-9, was propagated in MARC-145 cells in DMEM containing 2% FBS. Confluent MACR-145 cells were inoculated with PRRSV at multiplicity of infection (MOI) of 0.1 for 1 h at 37 °C with 5% CO_2_. Unattached viruses were removed by washing with phosphate-buffered saline (PBS). The infected cells were then incubated in fresh media for various time periods. After infection, PRRSV-infected MARC-145 cells were cultured for the required incubation period followed by freeze/thaw cycles. The supernatant was collected and PRRSV titer was determined by tissue culture infectious dose 50 (TCID50) or plaque assay by inoculating the supernatant on a monolayer of MARC-145 cells.

### Antigen uptake assay

For antigen uptake, MDCs (5 × 105) were treated with FITC ovalbumin (10 μg/mL; Thermo, USA) for 30 min at 37 °C. Non-specific binding was assessed by incubating MDCs on ice for 30 min. The cells were washed three times with icecold PBS containing 2% FBS. Subsequently, antigen uptake analyses were performed on a fluorescence-activated cell sorter (FACS) Canto II flow cytometer using FACSDiva software (BD Biosciences, USA), and analyzed using FlowJo software (TreeStar, USA).

### Quantitative PCR

Total RNA was isolated using TRIzolTM reagent (Thermo), in accordance with the manufacturer’s instructions, and reverse-transcribed to generate complementary DNA (cDNA) using oligo-dT primers shown in Table 1 (Bioneer, USA). Real-time qRT-PCR was performed using a StepOne Plus real-time PCR system (Applied Biosystems, USA). PCR was conducted in a 96-well reaction plate using 9 μL of SYBR green PCR Master Mix (Thermo). Target gene expression was normalized to the mRNA level of *β*-actin. Relative gene expression was calculated using the 2^−ΔΔCt^ method. For visualization, most datasets are presented as fold change (2^−ΔΔCt^), whereas selected datasets (Figure 5A; Figure 6B) are shown as log_2_-transformed values (−ΔΔCt), corresponding to log_2_ fold change.

**Table 1:** Primer sequences used for quantitative real-time PCR.

Target	Primer	Sequence (5' to 3')	Size, bp	Target	Primer	Sequence (5' to 3')	Size, bp
β-actin	Forward	GATGAGATTGGCATGGCTTT	122	IL-10	Forward	GTAATGCCGAAGGCAGAGAG	155
	Reverse	CACCTTCACCGTTCCAGTTT			Reverse	GCACTCTTCACCTCCTCCAC	
Zbtb46	Forward	CCTTCGTTGCTGCTAACTCC	201	HSPG2	Forward	CAGCGGAGCCAGAGTGTTC	82
	Reverse	AACGAGCCAAAGGAGAGACA			Reverse	CCAGGGTGTAAGCAGGAGAC	
CCL22	Forward	CCCTCGTCCTCCTTGCTAT	196	MYH9	Forward	GAGCAGAGCGAGGAGAAGAA	163
	Reverse	CAGATCTCTCGGTCCCTCAA			Reverse	TCTTGTTGGCTGAGTCGATG	
MMP12	Forward	TATGGACCCCCAGAAAAACA	146	VIM	Forward	TGAATACCAAGACCTGCTGA	93
	Reverse	TGCTTCCACCAGAAGAACCT			Reverse	GAAATCCTGCTCTCCTCTCC	
CCR7	Forward	TTGGTGAGAGCTAGGCTGGT	195	CD163	Forward	AAATTGCAAAGAGCCGAGAA	189
	Reverse	GATTCCGAGAGTTGGTTGGA			Reverse	CCCGGTATTGAATTTGATGG	
CD226	Forward	AGATGACACAGGGGAGGATG	177	PU.1	Forward	TCCCCCCTCAGCCATCA	63
	Reverse	TATTCGGTCAAGCGGTATCC			Reverse	GCGTTTGGCGTTGGTAGAGA	
Mertk	Forward	CTTGCTCTACTCCCGACTGG	210	C/EBPa	Forward	CTGGAGCTGACCAGTGACAA	211
	Reverse	TCGCCGCTGTAAATCTTCTT			Reverse	TGAGATCTGGAGACCCGAAA	
Marco	Forward	CTGCAAATCCTGCAAACTCA	129	Ets-2	Forward	CAAGCGAGAAGGACAGAACC	205
	Reverse	CTCTCCCTTGATTCCTGGTG			Reverse	ACCCCACGTAAAACAAGCTG	
CCL2	Forward	TCTCCAGTCACCTGCTGCTA	210	JUN	Forward	CCAAGATCCTGAAGCAGAGC	214
	Reverse	AGGCTTCGGAGTTTGGTTTT			Reverse	TTCTTGGGGCATAGGAACTG	
VSIG4	Forward	TTTTTCTCAGGGGACTGTGG	219	FOS	Forward	ACTACGAGGCGTCATCATCC	186
	Reverse	ACACACCTCGTGCCTATTCC			Reverse	GGCTGGTCGAGATAGCAGTC	
TNF-α	Forward	CCACCAACGTTTTCCTCACT	151	ORF6	Forward	GTACATTCTGGCCCCTGCCC	668
	Reverse	CCCAGGTAGATGGGTTCGTA			Reverse	GCCCTAATTGAATAGGTGAC	
IL-6	Forward	ATGGCAGAAAAAGACGGATG	215	ORF7	Forward	AAACCAGTCCAGAGGCAAGG	250
	Reverse	GTGGTGGCTTTGTCTGGATT			Reverse	GCAAACTAAACTCCACAGTGTAA	

### Flow cytometry

Cells were incubated with antibodies in PBS containing 1% FBS at 4 °C for 20 min. Fluorochrome-labeled monoclonal antibodies (mAbs) against porcine SLA-1 (JM1E3), SLA-2 (2E9/13), CD169 (3B11/11), CD163 (2A10/11), CD8 (76-2-11), CD45 (MIL13) (Bio-Rad Laboratories, USA); CD172a (15H6) (Southern Biotech, USA); CD3ε (BB23-8E6-8C8) and CD4 (74-12-4) (BD Biosciences); human CD209 (polyclonal; R&D Systems); mouse CD80 (16-10A1; BD Biosciences); ETS-2 (polyclonal; MyBioSource, USA); phosphor-ETS-2 (Polyclonal; Biorbyt, UK); and c-FOS (9F6; Cell Signaling, USA) were used. For secondary staining, cells labeled with primary antibodies were stained with the following antibodies: streptavidin BV605, FITC antimouse IgG1, APC anti-mouse IgG2a, PerCP anti-mouse IgG2b, and FITC anti-goat IgG. Fixable viability stain (BD Biosciences) was used to distinguish live and dead cells. Samples were acquired on a FACS Canto II flow cytometer using FACSDiva software, and analyzed using FlowJo.

### Confocal immunofluorescence microscopy

PRRSV infected cells were fixed with PBS containing 4% formaldehyde for 15 min at room temperature (RT), and then placed in a cytospin at 400 ×*g* for 10 min. Then, cells were permeabilized with 0.5% Triton X-100 in PBS for 3 min at RT, followed by blocking with 10% FBS for 30 min at RT. Cells were incubated with mouse antipig CD163 (Bio-Rad) and rabbit anti-PRRSV M protein antibody (Thermo), then stained with goat anti-rabbit IgG conjugated with Alexa Fluor 488 (BD Biosciences), goat anti-mouse IgG conjugated with Alexa Fluor 594 (Thermo), and DAPI for nuclei (Immunobioscience, USA). Images were captured using a laser scanning confocal microscope (LSM700; Carl Zeiss, Germany).

### ELISA

Plasma samples were collected from pigs; aliquots were prepared and stored at −20 °C until PRRSV-induced IL-4 levels were measured using a porcine IL-4 enzyme-linked immunosorbent assay (ELISA; R&D Systems), in accordance with the manufacturer’s instructions. The optical density of each well was measured at 450 nm using a VersaMax ELISA Microplate Reader (Molecular Devices, USA).

### Autologous T cell co-culture

Round-bottomed 96-well plates (Greiner Bio-One, Germany) were coated with 1.5 μg/mL of anti-pig CD3 mAbs (clone PPT7, IgG1). Free mAbs were removed by washing the wells three times with PBS. Differentiated MDCs were transferred into either CD3coated or non-coated plates. Then, MDCs were either mock-infected or infected with PRRSV at an MOI of 0.1 for 1 h. In parallel, autologous PBMCs were thawed and labeled with a proliferation dye, CellTraceTM Violet cell proliferation Kit (Life Technologies, USA). MDCs and violet-labeled PBMCs were seeded at a ratio of 1:10 in CD3-coated plates, then co-cultured for 3 days at 37 °C with 5% CO_2_.

### Lung tissue collection

For *in vivo* characterization of immune cells in PRRSV-infected pigs, the caudal left lung lobe was obtained from 7-to-11-week-old Landrace–Yorkshire–Duroc pigs (CJ F&C Co., Ltd., Seoul, Korea). Next, a 1-cm slice of external lung parenchyma was dissected, minced, and incubated for 2 h at 37 °C in complete RPMI 1640, supplemented with 100 IU/mL penicillin, 100 mg/mL streptomycin, 2 mM L-glutamine, and 10% inactivated FBS, containing 2 mg/mL collagenase D (Roche, Switzerland) and 0.1 mg/mL DNase I (Roche). Cells were passed through 100-μm cell strainers and red blood cells were lysed with erythrocyte lysis buffer (10 mM NaHCO_3_, 155 mM NH_4_Cl, and 10 mM EDTA).

### Statistical analyses

Data are presented as means ± standard deviations. Student’s *t*-test or one-way analysis of variance was performed to assess the statistical significance of differences between groups using GraphPad Prism (version 7.03, GraphPad Software, USA). *p*-values < 0.05 were considered statistically significant.

## Results

### Phenotypic and functional differences between MDCs^w/o IL-4^ and MDCs^with IL-4^

**Figure 1:**
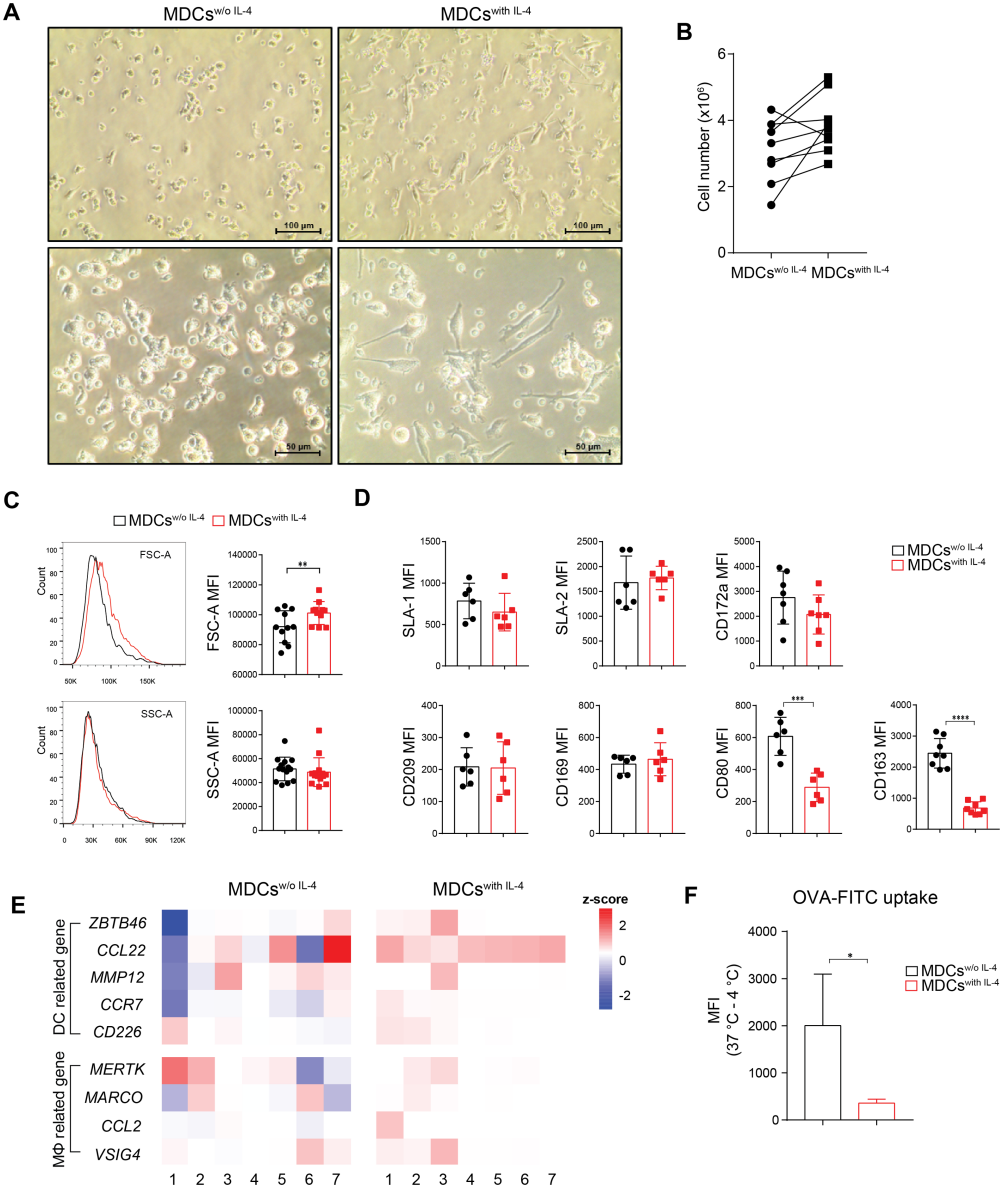
Porcine CD14^+^ monocytes (2 × 10^6^ cells/mL) were treated with GM-CSF (10 μg/mL) with (MDCs^with IL-4^) or without IL-4 (25 μg/mL, MDCs^w/o IL-4^) for 4 days to induce differentiation into MDCs. **(A)** Microscopy images show MDCs^w/o IL-4^ and MDCs^with IL-4^ morphologies. Bar = 100 μm (upper) or 50 μm (lower). **(B)** Absolute numbers of MDCs^w/o IL-4^ (●) and MDCs^with IL-4^ (■) are shown (*n* = 9, each line represents a different sample). **(C)** FSC-A and SSC-A levels of MDCs^w/o IL-4^ and MDCs^with IL-4^ (*n* = 11). One representative figure (left panel) from at least nine independent results (right) is shown. **(D)** MFIs of each cell surface marker (SLA-1, SLA-2, CD172a, CD209, C169, CD80, and CD163) were analyzed using flow cytometry and compared between MDCs^w/o IL-4^ and MDCs^with IL-4^ (*n* = 6). **(E)** mRNA expression levels of dendritic cell-associated molecules (*ZBTB46, CCL22, MMP12, CCR7,* and *CD226*) and macrophage-associated molecules (*MERTK, MARCO, CCL2,* and *VSIG4*) in MDCs^w/o IL-4^ and MDCs^with IL-4^ were analyzed using real-time qRT-PCR and normalized using *β*-actin (*n* = 7). Results are shown using a heat map. Color scale represents Z-score–normalized expression values. **(F)** Monocytes and the two types of MDCs were incubated with OVA-FITC (10 μg/mL) for 30 min at 37 °C. Nonspecific binding was assessed by incubating MDCs with the markers for 30 min at 4 °C. MFI minus the non-specific binding value of FITC on monocytes and the two types of MDCs are represented (*n* = 3). GM-CSF: Granulocyte-macrophage colony-stimulating factor; MDC: monocyte-derived cell; IL-4: interleukin-4; FSC-A: forward-scattered light-area; SSC-A: side scattered light-area; MFI: mean/median fluorescence intensity; OVA: ovalbumin; FITC: fluorescein isothiocyanate.

Porcine monocytes can be differentiated with GM-CSF to produce moMacs (Pakon et al., 2009) or GM-CSF and IL-4 to produce moDCs (Bautista et al., 2007). These cells are collectively known as MDCs. In the present study, two types of MDCs were compared: monocytes differentiated with GM-CSF (hereafter MDCs^w/o IL-4^) and GM-CSF together with IL-4 (hereafter MDCs^with IL-4^). To examine phenotypic properties in MDCs^w/o IL-4^ and MDCs^with IL-4^, cell number, morphology, and surface markers were compared after differentiation. The morphologies of the two cell types were clearly distinct (Figure 1A), although there was no difference in absolute cell number (Figure 1B). MDCs^w/o IL-4^ had a spherical shape with short hairy protrusions on the surface. In contrast, MDCs^with IL-4^ exhibited an elongated form with abundant dendrites (Figure 1A). The forward-scattered light-area (FSC-A) representing cell size was larger in MDCs^with IL-4^ than in MDCs^w/o IL-4^; there was no difference in side-scattered light-area (SSC-A) representing cell granularity (Figure 1C). The levels of CD80 (co-stimulatory molecule) and CD163 (macrophage lineage marker) were significantly lower in MDCs^with IL-4^ than in MDCs^w/o IL-4^; there were no differences in SLA-1, SLA-2, CD172a (myeloid lineage marker), CD209 (DC-SIGN; ctype lectin receptor), or CD169 (sialoadhesin or siglec-1; corresponding to integrin) (Figure 1D). Monocyte differentiation in cells with macrophage or DC characteristics is induced by the influence of specific growth factors and cytokines (Randolph et al., 2008). Thus, the profiles of DC- and macrophage-related genes were compared. As expected, the results showed that the levels of DC-related genes were higher in MDCs^with IL-4^ than in MDCs^w/o IL-4^ (Figure 1E). A notable functional difference between DCs and macrophages is their endocytosis ability (Autenrieth et al., 2007; Burgdorf et al., 2007). We found that ovalbumin uptake was significantly higher in MDCs^w/o IL-4^ than in MDCs^with IL-4^ (Figure 1F). Collectively, MDCs differentiated by GM-CSF with IL-4 exhibited phenotypic and genetic features consistent with dendritic cell differentiation, in contrast to MDCs differentiated with GM-CSF alone.

### PRRSV susceptivity in MDCs^w/o IL-4^ and MDCs^with IL-4^

**Figure 2:**
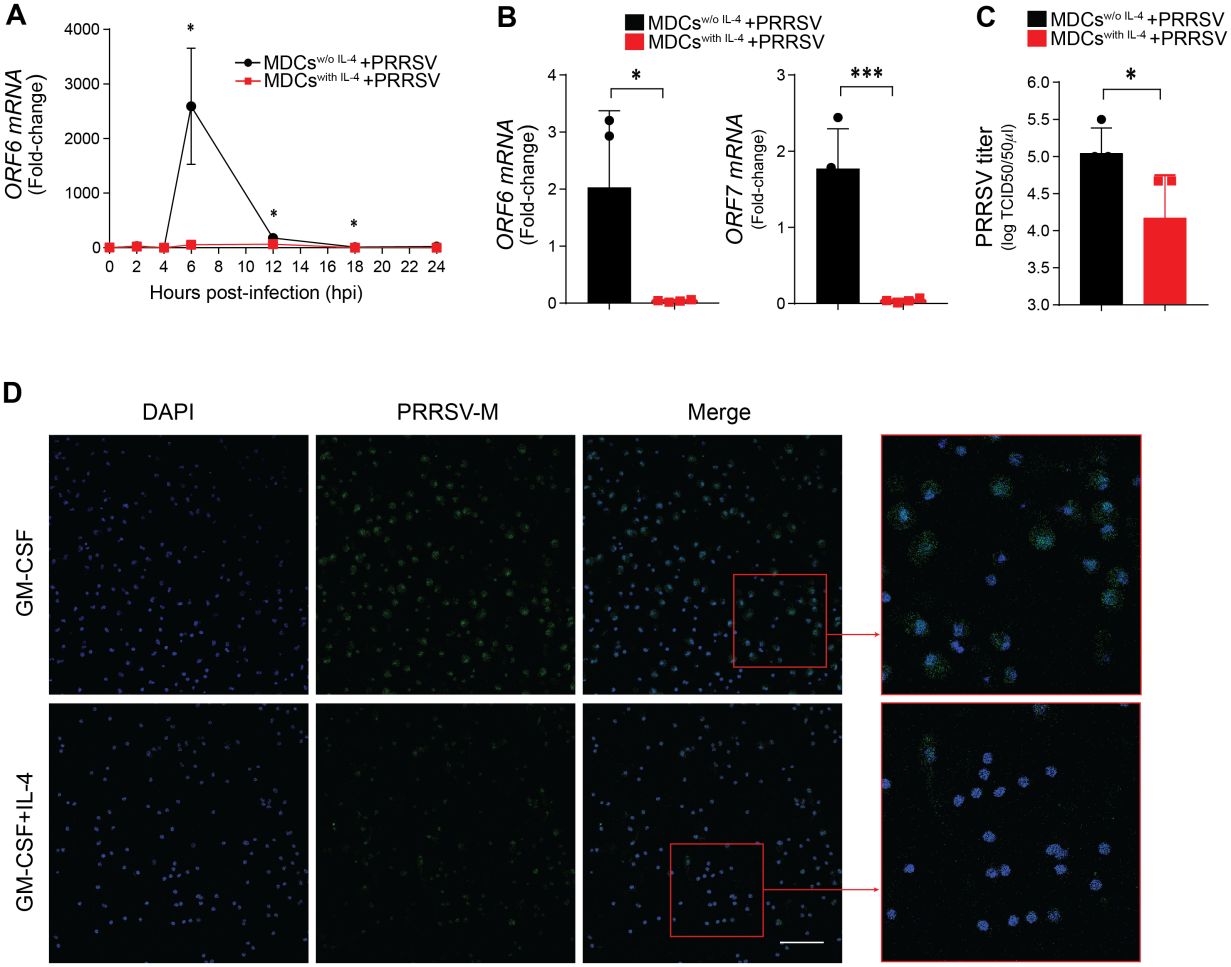
Porcine CD14^+^ monocytes were treated with GM-CSF with (MDCs^with IL-4^) or without IL-4 (MDCs^w/o IL-4^) for 4 days to induce differentiation. The two types of MDCs (2 × 10^6^ cells/mL) were infected with PRRSV (MOI 0.1) for 1 h at 37 °C with 5% CO_2_. Then, cells were washed with PBS and cultured in DMEM with 2% FBS. **(A)** PRRSV ORF6 mRNA expression levels were analyzed at 0, 2, 4, 6, 12, 18, and 24 hpi using real-time qRT-PCR and normalized using β-actin (*n* ≥ 15). **(B)** PRRSV ORF6 and ORF7 mRNA expression levels were analyzed at 6 hpi using real-time qRT-PCR and normalized using β-actin (*n* ≥ 4). **(C)** Viral titer was estimated using TCID50 at 72 hpi. **(D)** PRRSV M protein expression patterns were observed using confocal immunofluorescence microscopy. Bar = 50 μm. PRRSV: porcine reproductive and respiratory syndrome virus; PBS: phosphate-buffered saline; DMEM: Dulbecco’s modified eagles medium.

A hallmark of MDCs in pigs is their susceptivity to PRRSV. PRRSV has limited cellular tropism that exclusively involves the monocyte/ macrophage lineage, but it excludes DCs (Mair et al., 2014). The results of previous studies have indicated that MDCs^with IL-4^ exhibit phenotypic characteristics consistent with dendritic cell differentiation, compared with MDCs^w/o IL-4^; these findings suggest that PRRSV susceptivity also differs between the two MDC types. Therefore, in this study, PRRSV susceptivity was compared by measuring viral ORF6 mRNA expression for up to 24 h post-infection (hpi). The results showed that ORF6 mRNA expression was lower in MDCs^with IL-4^ than in MDCs^w/o IL-4^ at 6, 12, and 18 hpi (Figure 2A). ORF7 expression was also lower in MDCs^with IL-4^ than in MDCs^w/o IL-4^ after PRRSV infection (Figure 2B).

Furthermore, the use of a tissue culture infectious dose of 50% (TCID50) revealed that viral titers were significantly lower in MDCs^with IL-4^ than in MDCs^w/o IL-4^ (Figure 2C). As expected, PRRSV M protein levels were lower in MDCs^with IL-4^ than in MDCs^w/o IL-4^ after PRRSV infection (Figure 2D).

### PRRSV reactivity in MDCs^w/o IL-4^ and MDCs^with IL-4^

**Figure 3:**
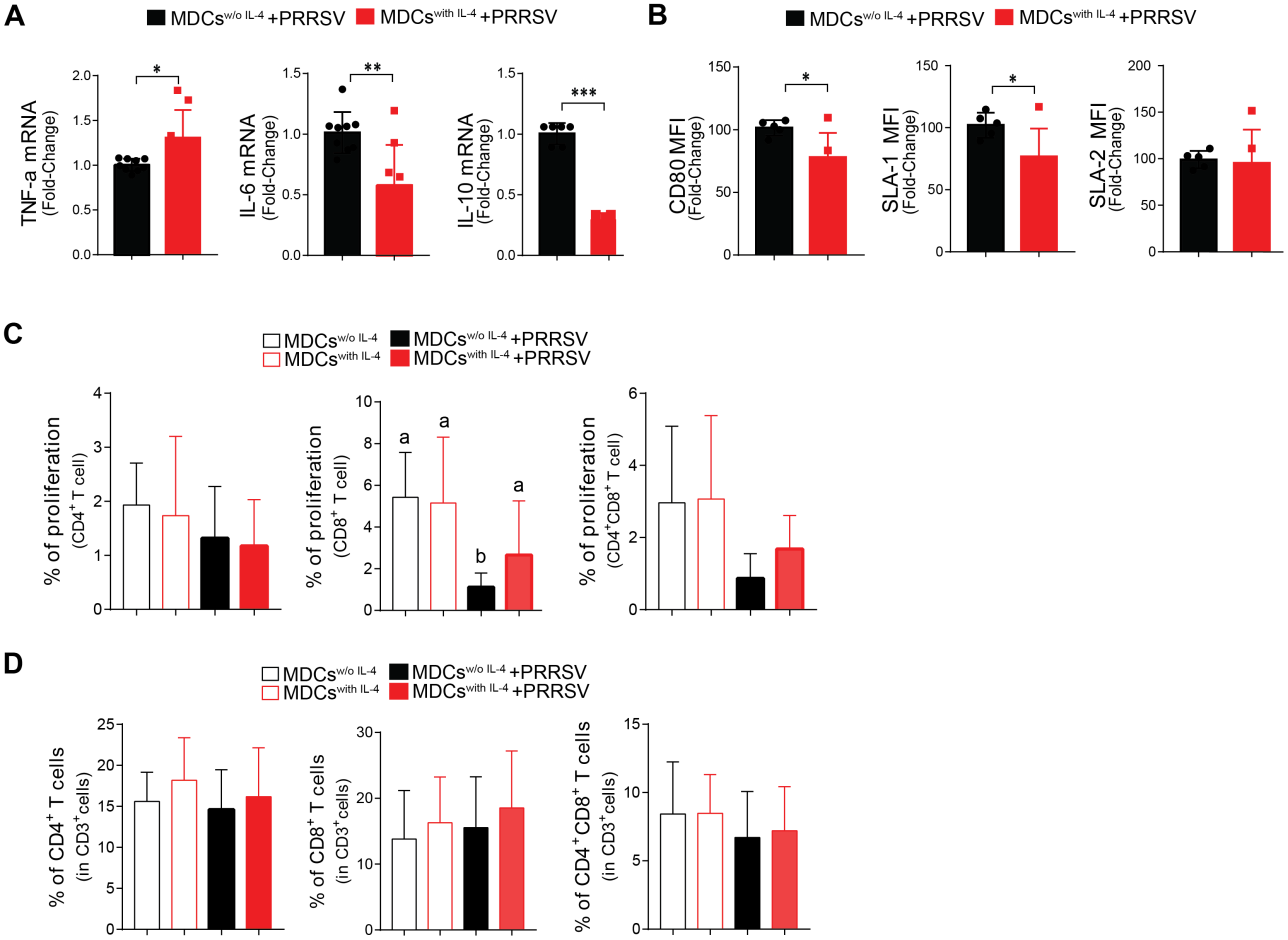
MDCs^with IL-4^ and MDCs^w/o IL-4^ were infected with PRRSV for 1 h at 37 °C. The cells were washed with PBS and cultured for an additional 24 h. Next, **(A)** mRNA expression levels of inflammatory cytokines (TNF-*α*, IL-6, and IL-10) were analyzed using real-time qRT-PCR and normalized using β-actin (*n* = 5). **(B)** CD80, SLA-1, and SLA-2 were analyzed using flow cytometry (*n* = 3). MDCs^with IL-4^ and MDCs^w/o IL-4^ were infected with PRRSV for 1 h and washed with PBS. CTV-labeled autologous PBMCs were mixed (MDCs: PBMCs = 1:10) and co-cultured for 3 days. Cells were stained with anti-pig CD3, CD4, CD8, and γδ TCR antibodies. **(C)** Proportions of CD4^+^, CD8^+^, and CD4^+^CD8^+^ T cells and **(D)** percentages of CTV-negative proliferated CD4^+^, CD8^+^, and CD4^+^CD8^+^ T cells were analyzed by flow cytometry (*n* = 9). Different lowercase letters (e.g., “a,” “b”) indicate statistically significant differences (*p* < 0.05) between groups. TNF-α: tumor necrosis factor alpha; CTV: Cell Trace™ Violet; PBMC: peripheral blood mononuclear cell.

The innate immune response after PRRSV infection involves the activation of cytokines and co-stimulatory molecules. To further investigate cytokine production, expression levels of tumor necrosis factor alpha (TNF-*α*), IL-6, and IL-10 were measured in MDCs^w/o IL-4^ and MDCs^with IL-4^ that had been infected with PRRSV for 24 h. TNF-α expression was significantly higher in PRRSV-infected MDCs^with IL-4^ than in PRRSV infected MDCs^w/o IL-4^; IL-6 and IL-10 expression levels were significantly lower in PRRSV-infected MDCs^with IL-4^ than in PRRSV-infected MDCs^w/o IL-4^ (Figure 3A; Supplementary Figure S1). Furthermore, the expression levels of CD80 and SLA-1 were significantly lower in MDCs^with IL-4^ than in MDCs^w/o IL-4^; there was no difference in SLA-2 expression (Figure 3B). Data from autologous T cell co-cultures have suggested that PRRSV-infected MDCs inhibit T cell proliferation (Nedumpun et al., 2019). To investigate the effect of PRRSV on T cell proliferation, Cell Trace ™ Violet-labeled peripheral blood mononuclear cells were co-cultured with PRRSV-infected MDCs^w/o IL-4^ or PRRSV-infected MDCs^with IL-4^. The proliferation of CD8^+^ T cells was significantly reduced during co-culture with PRRSV-infected MDCs^w/o IL-4^; minimal or no change was observed with PRRSV infected MDCs^with IL-4^ (Figure 3C center).

There were no differences in the percentages of co-cultured CD4^+^, CD8^+^, or CD4^+^CD8^+^ T cells (Figure 3D). These results suggest that IL-4-mediated regulation of monocyte differentiation reduces susceptivity to PRRSV infection by inhibiting CD8^+^ and CD4^+^CD8^+^ T cell proliferation.

### Receptor-mediated endocytosis in MDCs^w/o IL-4^ and MDCs^with IL-4^

**Figure 4:**
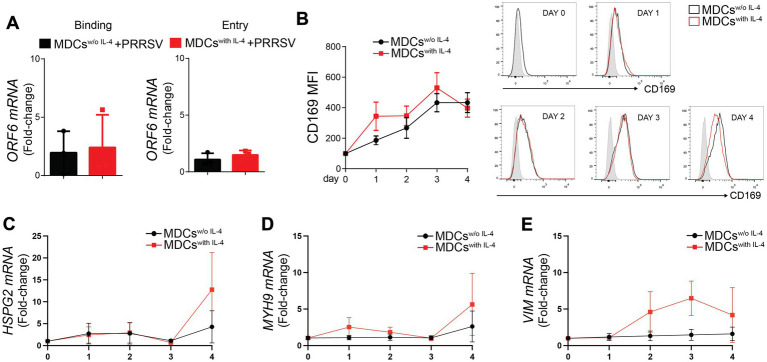
**(A)** MDCs^with IL-4^ and MDCs^w/o IL-4^ were infected with PRRSV for 1 h at 4 °C for binding analysis and for 1 h at 37 °C for entry analysis. After cells had been washed with PBS, mRNA expression levels of PRRSV ORF6 were analyzed using real-time qRT-PCR and normalized using β-actin (*n* = 3). **(B)** CD169 protein expression levels were measured at 0, 1, 2, 3, and 4 days after GM-CSF with (MDCs^with IL-4^) or without IL-4 (MDCs^w/o IL-4^) treatment using flow cytometry. One representative result of at least three independent experiments is shown (*n* = 3). **(C–E)** mRNA expression levels of heparin sulfate proteoglycan 2 (HSPG2), myosin heavy chain 9 (MYH9), and vimentin (VIM) were measured using real-time qRT-PCR and normalized using βactin > β-actin (*n* = 3).

Next, we examined how IL-4 regulates PRRSV susceptivity in MDC differentiation. The process of viral infection consists of four steps: receptor binding, entry, uncoating of the viral genome in the cytosol, and replication (Lunney et al., 2016). We first examined the receptor binding and entry of PRRSV in MDCs^w/o IL-4^ and MDC^with IL-4^. Binding was determined by culturing the cells with virus at 4 °C, and entry was evaluated by culturing at 37 °C. Although the viral titers differed between MDCs^w/o IL-4^ and MDCs^with IL-4^, viral binding and entry were similar in both cell types (Figure 4A). It has been reported that the binding of GP5, a viral glycoprotein, to CD169 (sialoadhesin) causes clathrin mediated endocytosis (Zhang and Yoo, 2015). Our results showed that CD169 expression gradually increased during the differentiation of monocytes into MDCs; the expression levels were similar in MDCs^w/o IL-4^ and MDCs^with IL-4^ (Figure 4B). Comparison of the mRNA expression levels of other PRRSV receptors—heparin sulfate proteoglycan 2 (Figure 4C), myosin heavy chain 9 (Figure 4D), and vimentin (Figure 4E)—revealed no differences between the two cell types during the differentiation process. Therefore, these results suggest that PRRSV susceptivity in MDCs^w/o IL-4^ and MDCs^with IL-4^ is independent of viral binding and entry.

### Regulation of viral uncoating in MDCs^w/o IL-4^ and MDCs^with IL-4^

**Figure 5:**
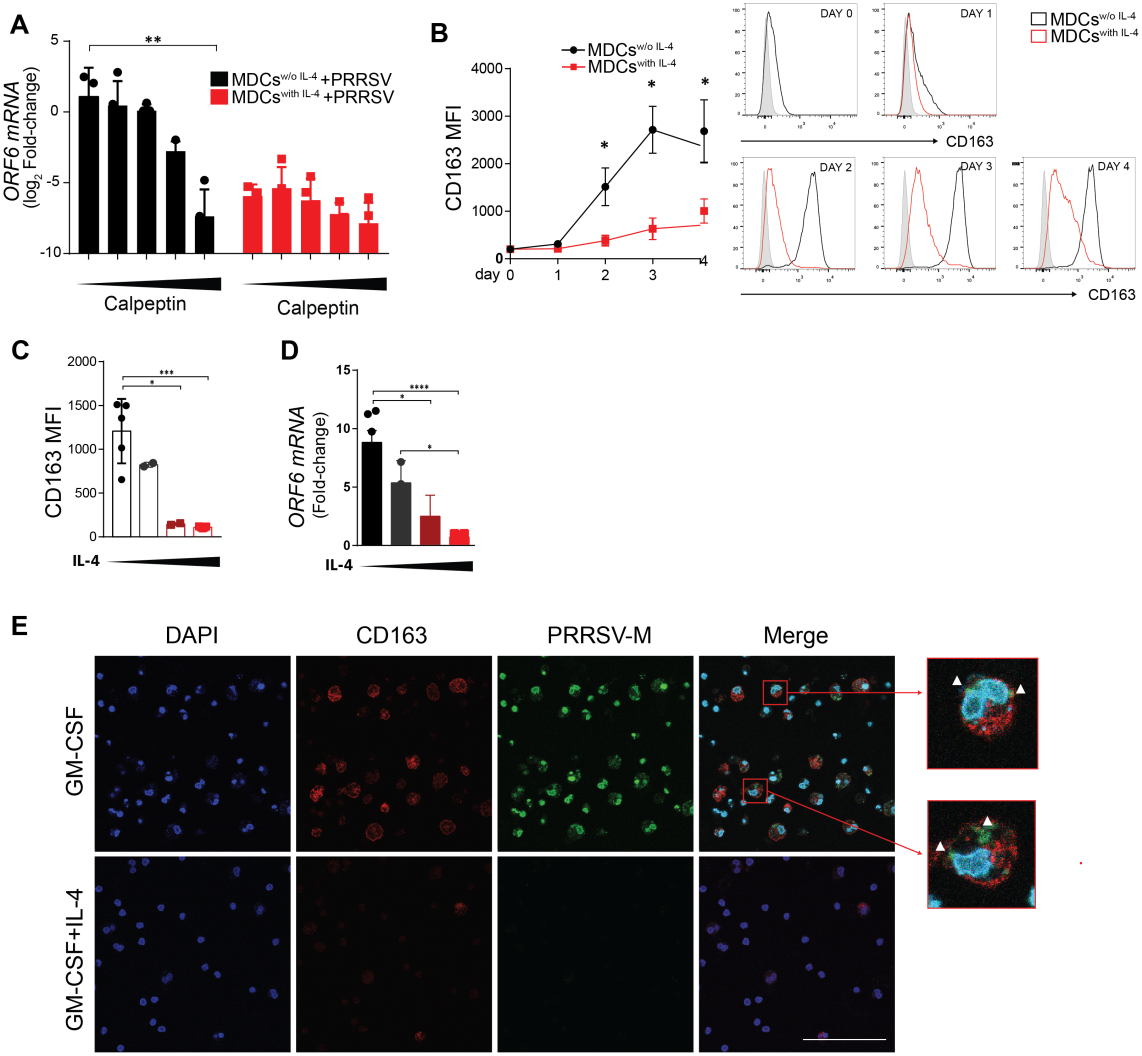
**(A)** MDCs^with IL-4^ and MDCs^w/o IL-4^ were infected with PRRSV for 1 h at 37 °C with 0, 20, 50, or 100 μM of calpeptin. After cells had been washed with PBS, they were cultured for an additional 6 h. Then, mRNA expression levels of PRRSV ORF6 were analyzed using real-time qRT-PCR and normalized using β-actin (*n* = 4). **(B)** *CD163 protein* expression levels were measured at 0, 1, 2, 3, and 4 days after GM-CSF with (MDCs^with IL-4^) or without IL-4 (MDCs^w/o IL-4^) treatment using flow cytometry. One representative result of at least three independent experiments is shown (*n* = 3). **(C,D)** *Mon*ocytes were treated with GM-CSF and 0, 0.25, 2.5, or 25 μg/mL of IL-4 for 4 days. Then, **(C)** CD163 protein expression levels were measured using flow cytometry. **(D)** Cells were infected with PRRSV for 1 h at 37 °C. After they had been washed with PBS, cells were cultured for an additional 6 h. Then, mRNA expression levels of PRRSV ORF6 were analyzed using real-time qRT-PCR and normalized using β-actin (*n* = 5). **(E)** CD163 and PRRSV M protein expression patterns were observed using confocal immunofluorescence microscopy. Bar = 50 μm.

After endocytosis, the viral genome undergoes an uncoating process (i.e., escape from the endosome into the host cell cytosol) (Burkard et al., 2018). Viral uncoating is mediated by the activity of calpain-1, which binds to the SRCR5 domain of CD163 (Yu et al., 2020). The effect of calpain-1 on PRRSV susceptivity in MDCs^w/o IL-4^ and MDCs^with IL-4^ was investigated by treatment with calpeptin, an inhibitor of calpain-1. Calpeptin regulated viral susceptivity in MDCs^w/o IL-4^ in a dose-dependent manner, whereas no noticeable effect was detected in MDCs^with IL-4^ (Figure 5A), suggesting that calpain-1-mediated viral uncoating contributes to PRRSV susceptivity in MDCs^w/o IL-4^, but not in MDCs^with IL-4^ conditions. Next, we examined the expression of CD163, a receptor involved in uncoating, in MDCs^w/o IL-4^ and MDCs^with IL-4^ during the differentiation step. Consistent with PRRSV susceptivity, CD163 expression was significantly greater in MDCs^w/o IL-4^ than in MDCs^with IL-4^ (Figure 5B). Additionally, CD163 expression was downregulated in an IL-4 dose-dependent manner (Figure 5C). PRRSV susceptivity also decreased in an IL-4 dose-dependent manner that paralleled CD163 expression (Figure 5D); CD163 and PRRSV M proteins displayed greater colocalization in MDCs^w/o IL-4^ than in MDCs^with IL-4^ (Figure 5E). These results suggested that the IL-4-mediated regulation of PRRSV susceptivity during MDC differentiation is dependent on viral uncoating driven by CD163.

### IL-4-mediated regulation of CD163 expression during MDC differentiation

**Figure 6:**
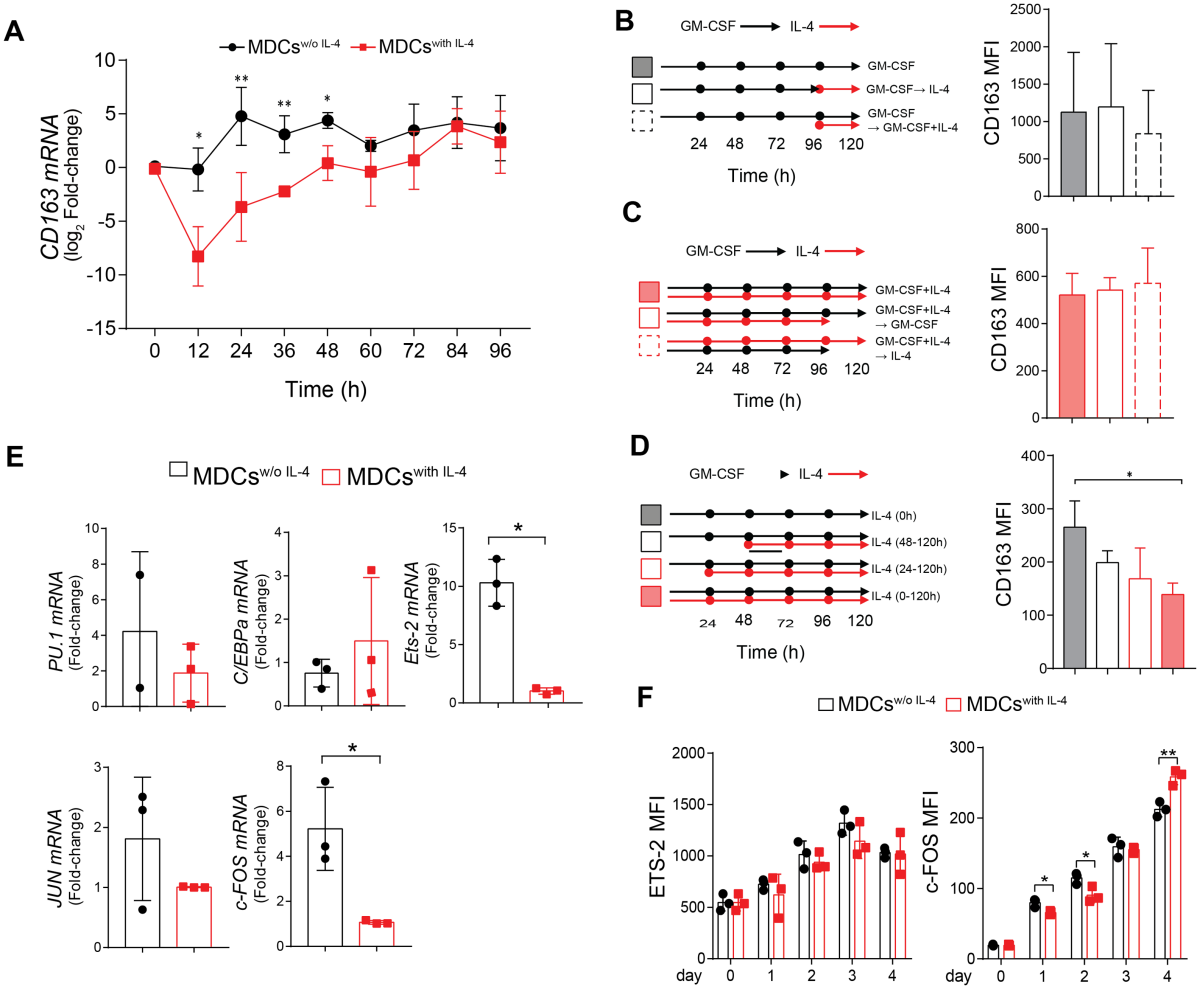
Porcine CD14^+^ monocytes were treated with GM-CSF with (MDCs^with IL-4^) or without IL-4 (MDCs^w/o IL-4^) for 4 days: **(A)** CD163 mRNA expression levels were analyzed using real-time qRT-PCR and normalized using β-actin at 0, 12, 24, 36, 48, 60, 72, 84, and 96 h after treatment (*n* = 5). **(B)** MDCs^w/o IL-4^ and **(C)** MDCs^with IL-4^ were harvested, washed with PBS, and cultured with media containing GM-CSF, IL-4, or GM-CSF with IL-4 for 24 h. Then, CD163 expression levels were measured using flow cytometry (*n* = 3). **(D)** Porcine CD14^+^ monocytes were treated with IL-4 at 0, 24, and 48 h after differentiation had been initiated with GM-CSF. CD163 protein expression was measured using flow cytometry (*n* = 3). **(E)** One day after treatment, CD163 transcription factor candidates, *PU.1, C/EBPα, Ets-2, JUN, and c-FOS* were analyzed by real-time qRT-PCR and normalized using βactin > β-actin (*n* = 3). **(F)** Protein expression levels of ETS2 and c-FOS were measured using flow cytometry at 0, 1, 2, 3, and 4 days after treatment (*n* = 3).

CD163 expression can be regulated in two ways: cleavage of membrane-bound CD163 (mCD163) into soluble CD163 (sCD163) via the A disintegrin and metalloproteinase domain-containing protein 10 (ADAM10) enzyme (Lemieux et al., 2007) or downregulation of CD163 gene expression. To examine whether mCD163 expression is downregulated by IL-4-mediated sCD163 cleavage during MDC differentiation, cells were treated with batimastat (an ADAM10 inhibitor) during the differentiation process. The results showed that the expression of CD163 was restored in MDCs via suppression of sCD163 cleavage (Supplementary Figure S2), indicating that IL-4-mediated downregulation of CD163 could be related to the involvement of sCD163 cleavage. Next, to investigate whether the difference in CD163 expression between MDCs^w/o IL-4^ and MDCs^with IL-4^ originates at the gene level, the mRNA expression levels of *CD163* in MDCs^w/o IL-4^ and MDCs^with IL-4^ were examined. *CD163* mRNA expression was significantly lower in MDCs^with IL-4^ than in MDCs^w/o IL-4^, as early as 12 h after the start of differentiation (Figure 6B); this finding suggests that *CD163* gene expression was inhibited by IL-4 in the early stage of differentiation. Next, we investigated whether treatment with GM-CSF or IL-4 induces CD163 expression in differentiated MDCs. As shown in Figures 6C, MDCs^w/o IL-4^ and MDCs^with IL-4^ cultured for 4 days were additionally treated with GM-CSF alone, IL-4 alone, or GM-CSF with IL-4. The results showed that CD163 expression remained unchanged after differentiation (Figures 6B,C), suggesting that IL-4 treatment regulates CD163 expression only in the early stage of MDC differentiation. Next, to determine the specific timing of effects on MDC differentiation, IL-4 was added at various time points: 0, 24, and 48 h after differentiation had been initiated in GM-CSF-treated MDCs (Figure 6E). A gradual decrease in CD163 expression was observed, corresponding to the time of IL-4 exposure (Figure 6E). Taken together, these results suggest that the regulation of CD163 expression is affected by IL-4 in the early stage, particularly on the first day, of differentiation. Our previous results showed that CD163 gene expression during MDC differentiation is regulated by IL-4 (Figure 6F); thus, the expression levels of CD163 transcription factor candidates, *PU.1, C/ EBPα, Ets-2, JUN,* and c-*FOS*, were examined in MDCs^w/o IL-4^ and MDCs^with IL-4^. The mRNA expression levels of *Ets-2* and *c-FOS* were lower in MDCs than in MDCs^w/o IL-4^ (Figure 6F). Consistent with these findings, the protein expression level of c-FOS was lower in MDCs on days 1 and 2 after initial differentiation; there was no difference in ETS-2 protein expression (Figure 6G). Thus, CD163 can be controlled via IL-4-mediated downregulation of c-FOS in the early stage of MDC differentiation.

### Validation of immune cell distribution and plasma IL-4 expression in the lungs of PRRSV infected pigs

**Figure 7:**
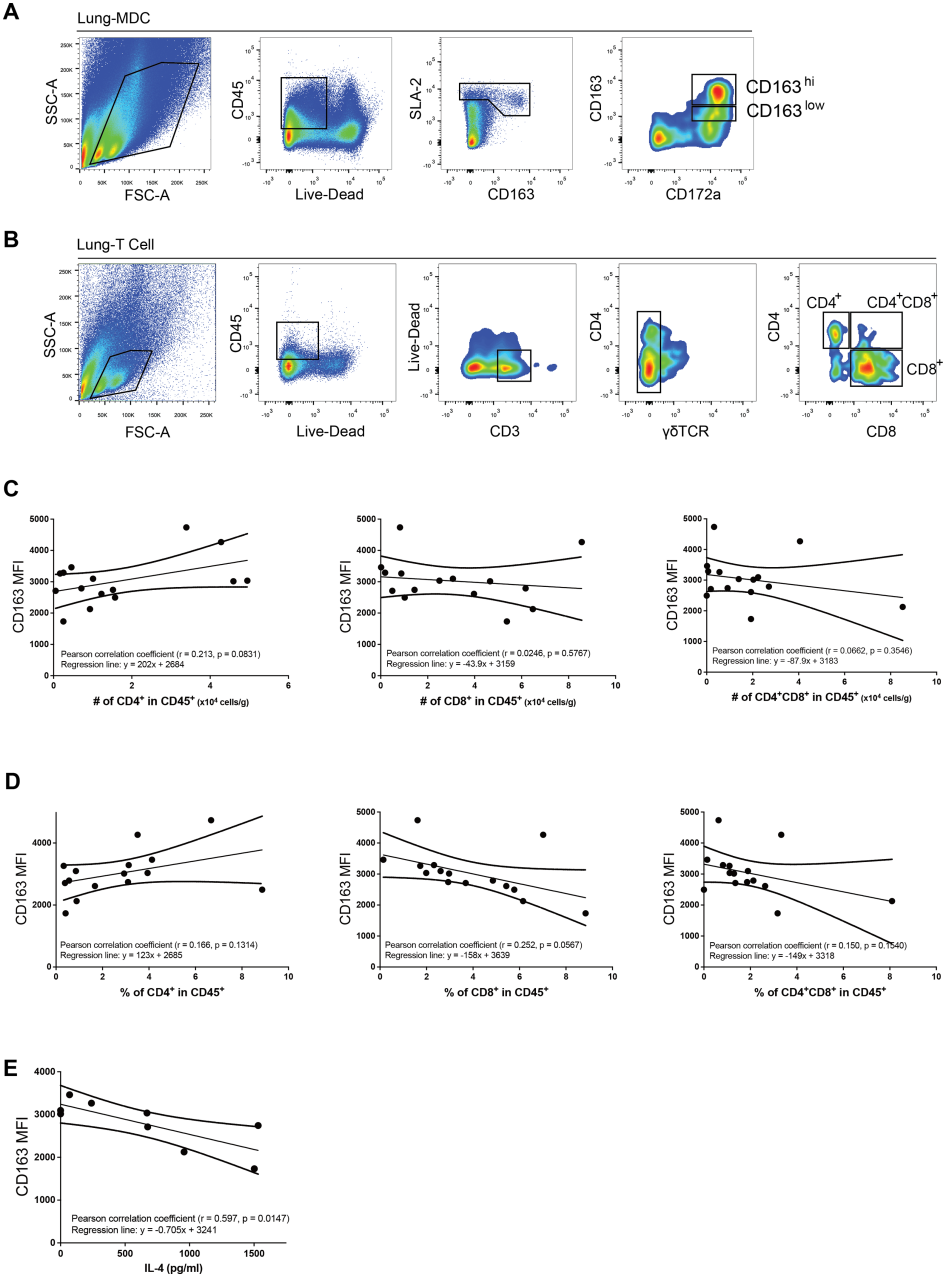
Caudal left lung lobes were obtained from 7-to-11-week-old pigs, as described in Materials and Methods. Dot plot gating strategy of lung tissue to isolate **(A)** MDCs and **(B)** T cells. **(C–E)** CD163 MFI on lung MDCs using flow cytometry. Correlation between CD163 MFI and **(C)** absolute number or **(D)** percentage of CD4^+^, CD8^+^, and CD4^+^CD8^+^ T cells were analyzed using the Pearson correlation coefficient (*R*) with two-tailed *p*-value (*n* = 15). **(E)** Plasma IL-4 levels were analyzed by ELISA. Correlation between plasma IL-4 and CD163 in MDCs was determined using the Pearson correlation coefficient (*r*) with two-tailed *p*-value (*n* = 9).

To further characterize the immune cell distribution *in vivo*, we examined lung cells that had been isolated from PRRSV-infected pigs. The results showed that CD163^low^ and CD163^hi^ MDCs were present in the lungs (Figure 7A), as described in a previous report (Bordet et al., 2018b). Furthermore, three T cell sub-populations were found in the lungs: CD4^+^, CD8^+^, and CD4^+^CD8^+^ T cells (Figure 7B). Next, to determine correlations between MDC and T cell distributions, the absolute number (Figure 7C) and proportion of T cells (Figure 7D) in lungs from PRRSV-infected pigs were compared via CD163 expression in MDCs. The results showed that the proportion of CD8^+^ T cells was negatively correlated with CD163 expression in MDCs (Figure 7D middle). This finding is similar to the outcome of the *in vitro* autologous T cell co-culture experiment (Figure 3C). It was intriguing that a higher concentration of plasma IL-4 led to lower CD163 expression in MDCs (Figure 7E). These results indicate that the expression of IL-4 in PRRSV-infected pigs could downregulate CD163 expression in MDCs.

## Discussion

This study investigated the effects of MDC differentiation with GM-CSF and IL-4 (MDCs^with IL-4^) or GM-CSF without IL-4 (MDCs^w/o IL-4^). The differentiation of MDCs from monocytes into MDCs^w/o IL-4^ and MDCs^with IL-4^ in humans results in heterogeneity (Sander et al., 2017). MDCs^w/o IL-4^ in humans show properties of both macrophages and DCs; however, MDCs^with IL-4^ contain fewer macrophages with more DC characteristics, compared with MDCs^w/o IL-4^. The present study revealed that, pig MDCs^with IL-4^ exhibit features consistent with dendritic cell differentiation, compare with pig MDCs^w/o IL-4^.

A previous report showed that human classical monocytes strongly express CD163, whereas porcine classical monocytes do not (Ziegler-Heitbrock, 2014), supporting the notion that CD163 regulation in monocyte lineage cells differs between humans and pigs. Indeed, whereas human studies have shown no differences in CD163 and CD80 between MDCs^w/o IL-4^and MDCs^with IL-4^, the levels of CD80 and CD163 are higher in pig MDCs^w/o IL-4^ than in pig MDCs^with IL-4^.

In PRRSV-infected pigs, IL-4 is present in the blood (approximately 600–1,000 pg./mL) at 2 days post-infection (Dwivedi et al., 2012). However, the IL-4-producing cells in PRRSV infected pigs have not been clearly identified; it is generally accepted that IL-4-producing cells in humans are basophils, eosinophils, mast cells, T helper 2 cells, T follicular helper cells, and natural killer T cells (Le Gros et al., 1990). Considering that IL-4 appears at an early time point during an infection, IL-4-producing cells are likely to be innate immune cells (rather than T or B cells). However, the precise cellular source of IL-4 and its temporal relationship with MDC differentiation *in vivo* are still not clearly defined. Therefore, the observed association between IL-4 levels and CD163 expression should be interpreted as correlative rather than causal.

In this study, we conducted co-culture experiments using MDCs and autologous T cells; we found that PRRSV-infected MDCs^w/o IL-4^ significantly suppressed CD8^+^ T cell proliferation, consistent with the notion that PRRSV infection can suppress T cell activity (Nedumpun et al., 2019). These results are likely due to the fact that cytokine-mediated effects, rather than antigen-presenting capacity itself, play a more dominant role in regulating CD8^+^ T cell proliferation in this context. As shown in the data, TNF-*α*, which promotes CD8^+^ T cell division, was reduced in MDCs^w/o IL-4^ compared to MDCs^with IL-4^ during PRRSV infection. In contrast, IL-6 and IL-10, which are known to suppress CD8^+^ T cell proliferation, were increased in MDCs^w/o IL-4^ relative to MDCs^with IL-4^ during PRRSV infection (Figure 5A). Similar cytokine expression trends were observed over multiple time points (Days 1–3), suggesting that these differences were maintained beyond the initial 24 h response. In addition, the co-culture system using anti-CD3 stimulation was not designed to directly evaluate antigen-presenting capacity itself. Furthermore, the expression patterns of antigen presentation- and co-stimulatory molecules (e.g., MHC class I and CD80) did not correlate with the observed CD8^+^ T cell proliferation responses (Figure 5B). Nevertheless, we recognize that additional experiments, including cytokine-neutralizing approaches, will be required to establish the precise mechanistic relationship between cytokine regulation and CD8^+^ T cell proliferation.

In addition, type I interferon (IFN) responses may contribute to the observed differences in CD8^+^ T cell proliferation. Type I IFNs are known to enhance CD8^+^ T cell activation and expansion during viral infection. Although IFN responses were not directly assessed in this study, the reduced viral replication observed in MDCs^with IL-4^ may be associated with altered innate antiviral signaling, which could indirectly influence CD8^+^ T cell responses. Further investigation will be required to clarify this potential link.

CD163, a receptor for PRRSV, is involved in the internalization of PRRSV into target cells, as well as PRRSV uncoating (i.e., escape from endosomes into the cytosol) (Burkard et al., 2017). The uncoating process may occur through the activity of calpain-1 (Yu et al., 2020), a neutral calcium-dependent cysteine protease (Goll et al., 2003). In MDCs^w/o IL-4^ with high CD163 expression, PRRSV susceptivity was significantly reduced by treatment with calpeptin, an inhibitor of calpain-1. However, in MDCs^with IL-4^ exposed to the same conditions, viral susceptivity was unaffected. These results suggest that calpain-1-mediated viral uncoating contributes to PRRSV susceptivity in MDCs^w/o IL-4^, whereas its role in MDCs^with IL-4^ remains unclear due to the low baseline level of infection. This finding emphasizes the possibility that the difference in PRRSV susceptivity between MDCs^w/o IL-4^ and MDCs^with IL-4^ is related to the CD163-mediated uncoating process.

Previous studies have suggested that PRRSV replication is influenced by autophagy (Ko et al., 2017) and mTOR signaling (Zhang et al., 2019) pathways. Modulation of autophagy has been shown to have an effect on PRRSV replication (Sun et al., 2012), and mTOR signaling is known to regulate both autophagy (Deretic and Levine, 2009) and cellular metabolism(Saxton and Sabatini, 2017). Given that IL-4 has been linked to metabolic reprogramming during MDC differentiation (Sander et al., 2017), it is plausible that IL-4 indirectly influences PRRSV replication through these pathways. However, these mechanisms were not directly examined in the current work and should be regarded speculative.

In addition, endo-lysosomal pathways may contribute to viral degradation, and CD163 has been implicated in regulating viral trafficking within host cells (Yu et al., 2020). Whether IL-4 mediated changes in CD163 expression affect these processes remains to be determined.

In the present study, IL-4-mediated downregulation of CD163 during MDC differentiation appears to involve both post-translational and transcriptional regulatory mechanisms. IL-4-induced ADAM10 activity promotes shedding of CD163, consistent with the observed reduction in membrane-bound CD163 (mCD163) and the increase in soluble CD163 (sCD163). A previous report demonstrated that ADAM10 in humans is activated by IL-4 in the U-937 monocyte cell line and primary cultures of B cells (Lemieux et al., 2007). In line with this, treatment with batimastat, an inhibitor of ADAM10, partially restored mCD163 expression in MDCs. However, this recovery was incomplete, suggesting that additional regulatory mechanisms contribute to CD163 downregulation.

Notably, CD163 mRNA expression was markedly reduced during the early phase of IL-4-mediated differentiation, whereas IL-4 treatment at later time points had minimal effects (Figures 2C,D). These findings indicate that transcriptional regulation significantly influences CD163 expression levels, likely mediated by transcription factors such as c-FOS. Taken together, these results suggest that IL-4 regulates CD163 expression through a combination of early transcriptional suppression and subsequent ADAM10-mediated cleavage, with transcriptional control likely being the predominant mechanism.

The *in vivo* samples were obtained under field conditions, and PRRSV infection was confirmed as North American-type (PRRSV-2) by an external diagnostic service (Optipharm, Korea). Documentation of the diagnostic results has been provided as Supplementary material. However, detailed metadata such as viral strain, dose, and exact time post-infection were not available due to source restrictions. Therefore, these findings should be interpreted as correlative observations.

Although the *in vivo* findings support the biological relevance of our *in vitro* observations, these data should be interpreted with caution. The *in vivo* analysis was performed using field-derived PRRSV-positive pigs without matched healthy controls, and therefore potential environmental or co-infection-associated effects cannot be fully excluded. Accordingly, the *in vivo* results were not intended to provide definitive mechanistic evidence, but rather supportive observational data associated with PRRSV-infected conditions. The interpretation of these findings was therefore made in conjunction with the controlled in vitro experiments.

PRRSV is transmitted through the respiratory and reproductive organs. When PRRSV infects the respiratory tract in pigs, invasion occurs via CD163 on alveolar macrophages (Burkard et al., 2017). However, it is unclear how PRRSV reaches alveolar macrophages to cause infection and disease. Similar to humans, the respiratory system in pigs is divided into upper and lower sections; the tonsils are located in the upper section. The tonsils contain populations of mononuclear phagocytes, including DCs, monocytes, and macrophages (Soldevila et al., 2018). Although the immune response to PRRSV infection in the tonsils has not been fully elucidated, macrophages in the tonsils express CD163 (Soldevila et al., 2018). Thus, PRRSV infection may initially occur in the tonsils, then spread to alveolar macrophages in the lower respiratory tract.

## Conclusion

**Figure 8:**
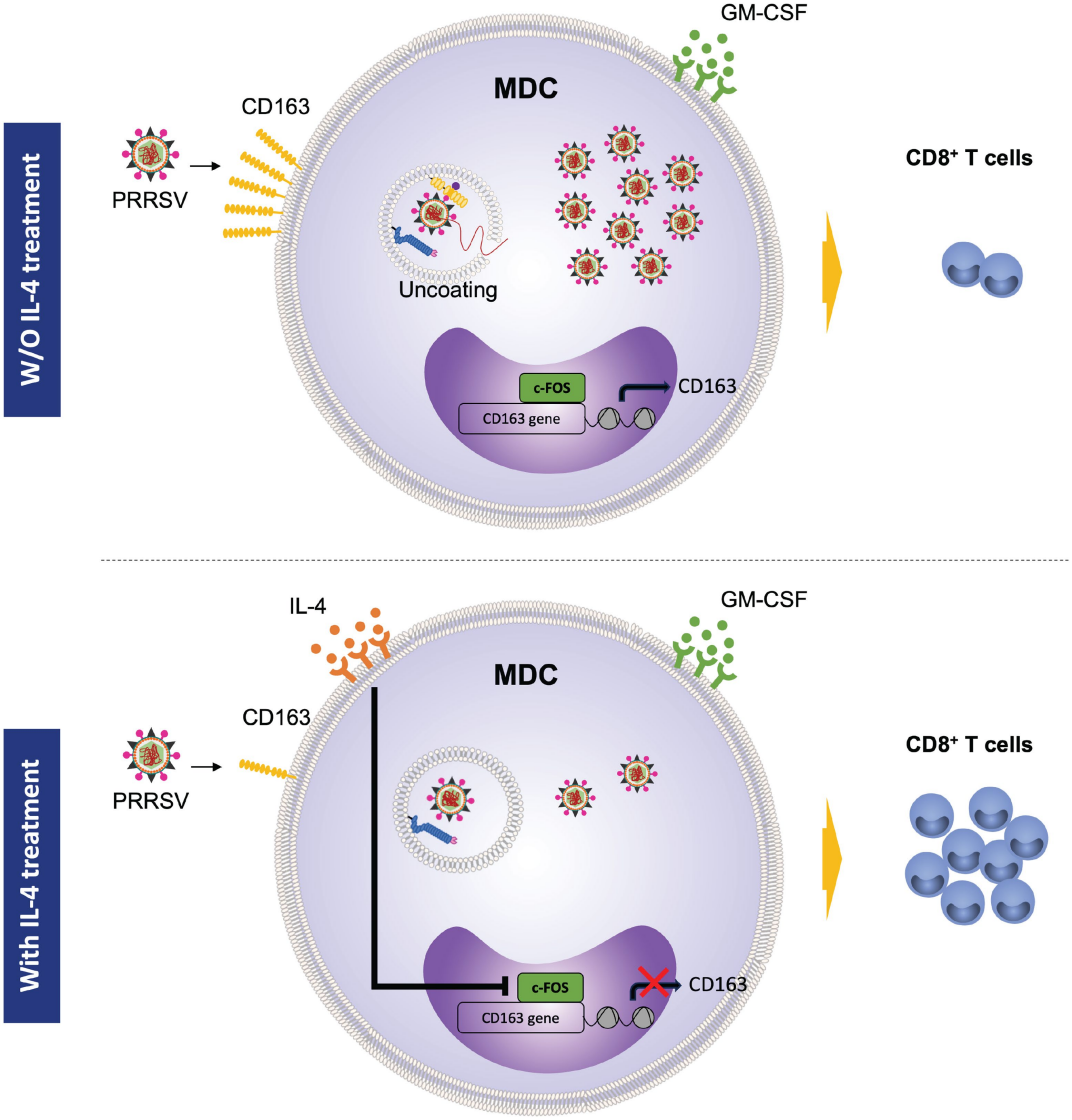
Graphical summary of this study. MCDs differentiated without IL-4 are highly susceptible to PRRSV. However, when IL-4 is introduced in the early stages of differentiation, the expression of c-FOS, which regulates CD163 expression, is suppressed. As a result, the expression of CD163, a receptor involved in virus uncoating, decreases, leading to a reduction in virus uncoating and inhibition of PRRSV infection. Additionally, the proliferation of CD8^+^ T cells, which was suppressed upon PRRSV infection, was restored. Furthermore, the analysis of infected pigs showed a negative correlation between the expression of CD163 in MDCs and both CD8^+^ T cells and IL-4.

We demonstrated that IL-4 production in PRRSV-infected pigs is associated with reduced CD163 expression. *In vitro,* IL-4 mediated MDC differentiation was accompanied by decreased PRRSV replication and increased CD8^+^ T cell proliferation. Furthermore, the proportion of CD8^+^ T cells was negatively correlated with CD163 expression in lung MDCs from PRRSV-infected pigs (Figure 8) and plasma IL-4 levels were also inversely correlated with CD163 expression in MDCs. Collectively, these findings suggest that IL-4 may contribute to host immune responses against PRRSV infection, although further studies are required to establish causal mechanisms.

## Data Availability

The original contributions presented in the study are included in the article/Supplementary material, further inquiries can be directed to the corresponding author.
