# Supplementary material for: IL-4-mediated monocyte differentiation modulates CD163 expression and PRRSV infection
Source: Front Microbiol. 2026 Jun 4;17:1817131. doi: 10.3389/fmicb.2026.1817131 (PMC13275479; doi:10.3389/fmicb.2026.1817131)
Supplement: Supplementary file 1 [file Data_Sheet_1.pdf]

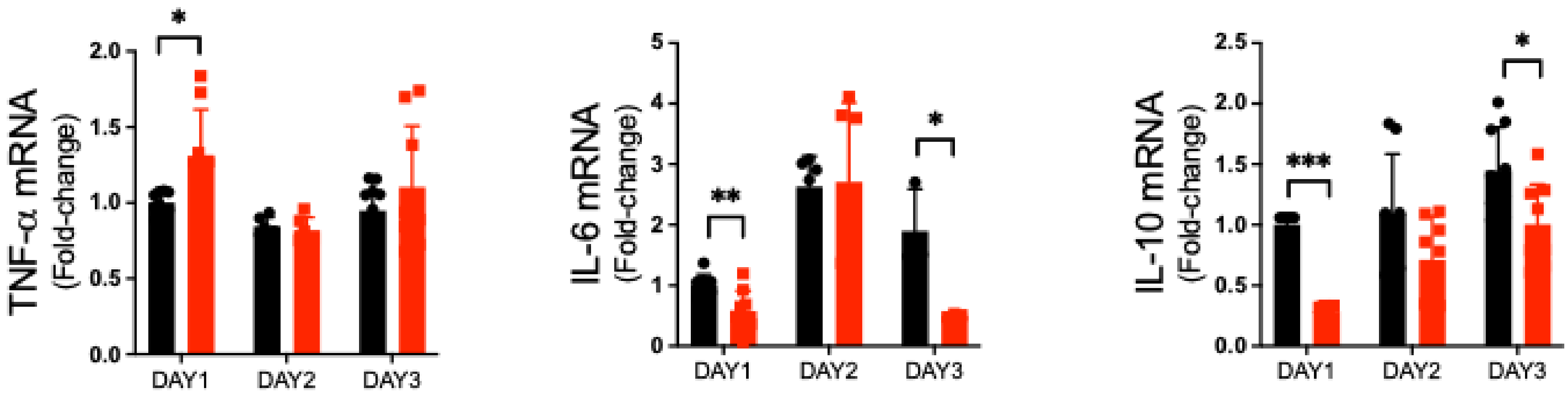

Supplementary Figure 1. MDCs $^{\text{with IL-4}}$ and MDCs $^{\text{w/o IL-4}}$ were infected with PRRSV for 1 h at 37°C. The cells were washed with PBS and cultured for an additional 1, 2, or 3 days. mRNA expression levels of inflammatory cytokines (TNF-α, IL-6, and IL-10) were analyzed using real-time qRT-PCR and normalized to β-actin (n = 5).

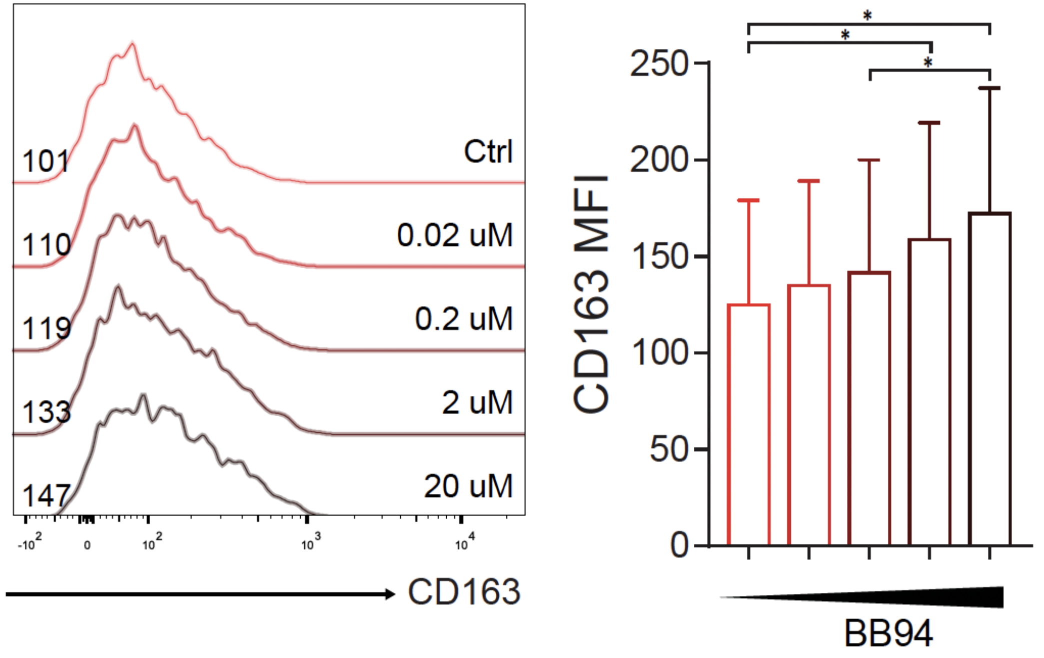

Supplementary Figure 2. Porcine CD14+ monocytes were treated with GM-CSF with (MDCs $^{\text{with IL-4}}$) or without IL-4 (MDCs $^{\text{w/o IL-4}}$) for 4 days (A); concurrently, ADAM10 activity was inhibited by 0, 0.02, 0.2, 2, or 20 μM of batimastat. CD163 expression was measured using flow cytometry (n = 3). ADAM10: A disintegrin and metalloproteinase domain-containing protein 10.
